# Temporal Change, Frequency, and Spatial Distribution of Mating Types of *Bipolaris maydis* in China Provide Indirect Evidence for Potential Sexual Reproduction

**DOI:** 10.3390/jof12080624

**Published:** 2026-08-19

**Authors:** Yuli Dai, Lin Gan, Xiaofei Liu, Chengzhong Lan, Yuanwen Guo, Xiujuan Yang

**Affiliations:** 1 Fujian Key Laboratory for Monitoring and Integrated Management of Crop Pests, Institute of Plant Protection, Fujian Academy of Agricultural Sciences, Fuzhou 350013, China; millergan@yeah.net (L.G.); lxf_sophie@126.com (X.L.); lczhong7911@126.com (C.L.); 2 State Key Laboratory for Biology of Plant Diseases and Insect Pests, Institute of Plant Protection, Chinese Academy of Agricultural Sciences, Beijing 100193, China; guoyuanwen@caas.cn

**Keywords:** *Bipolaris maydis*, southern corn leaf blight, mating types, heterothallism, sexual reproduction

## Abstract

The heterothallic fungus *Bipolaris maydis*, the causal organism of southern corn leaf blight, is a destructive pathogen of corn and is considered to predominantly reproduce asexually, as no perfect reproductive stage has been discovered under natural conditions. In this study, a multiplex PCR with mating type-specific primers was used to investigate the temporal change and spatial distribution of mating types in several *B. maydis* populations in China. Mating type monitoring demonstrated that both mating types co-existed throughout the entire monitoring period across the seven monitoring locations, with mating type frequencies skewing significantly (*p* < 0.05) to MAT1-2 during the period of 2015 to 2020 and being practically symmetric after 2020, implying that the two populations were approaching equilibrium. Both mating type isolates were detected within the same leaf or field, affording a high theoretical likelihood for the two mating type isolates to cross sexually. In addition, both MAT1-1 and MAT1-2 isolates were detected from large-scale geographical regions in China, with mating type frequencies approaching a null hypothesis ratio of 1:1 in most populations, suggesting frequency-dependent selection consistent with sexual reproduction. Conclusively, our results provided indirect evidence supporting the potential for sexual reproduction in *B. maydis* under natural conditions, although no sexual morphs have yet been discovered in the field.

## 1. Introduction

Corn (*Zea mays* L.) is one of the most widely cultivated food crops worldwide and is also grown for diverse applications [1,2]. It is grown across the northeast spring, Huang–Huai–Hai summer, and the south mountainous corn-growing regions in China [2,3]. During the 2024 cropping year, the total yield of corn was approximately 0.295 billion tons, accounting for 40% of the total grain output, with a total cultivated acreage and average yield of 44.8 million hectares and 6591 kg/ha, respectively [4,5]. The total yield, total cultivated acreage, and average yield of corn increased by 11.6%, 7.8%, and 4.1%, respectively, from 2020 to 2024 [4]. Nevertheless, corn production is continually threatened by southern corn leaf blight (SCLB) in most corn-growing regions of China. SCLB is a destructive foliar disease caused by *Bipolaris maydis* (Nisikado et Miyake) Shoem (Teleomorph: *Cochliobolus heterostrophus* (Drechsler) Drechsler) [6,7]. This fungal pathogen emerged with devastating consequences during the early 1970s in the U.S., when a novel physiological race T occurred, resulting in substantial economic losses of more than one billion dollars [8]. Subsequently, physiological races C and O of *B. maydis* were reported. Race C was first reported in China and has remained confined to the country [9,10]. Race O is the preponderant race distributed worldwide [8,11]. Despite there being several available control measures, such as chemical fungicide application, resistant material utilization, and crop rotation, that can weaken SCLB severity in the field, SCLB still causes substantial yield losses ranging from 10% to 68% [7,12]. Hence, *B. maydis* represents a major biotic restriction on corn yields in China.

In the heterothallic ascomycete *B. maydis*, sexual reproduction is regulated by a special mating type (MAT) locus with two alternative idiomorphs, designated MAT1-1 and MAT1-2 [13,14]. Traditional measures for mating type determination in heterothallic fungi involve pairing the mating type known isolate with a test isolate and monitoring the formation of sexual fruiting bodies (e.g., perithecia, asci, or ascospores) [15,16,17]. However, this approach involves a protracted procedure, is easily impacted by external factors (e.g., inductive media, nutritive materials, and environmental conditions), and frequently fails to produce the anticipated results owing to weak compatibility between isolates [16,18]. With the development of cloning and sequencing of MAT loci, DNA-based methods have been established to determine the mating types of numerous plant-pathogenic heterothallic fungi, including conventional PCR [19,20,21], multiplex PCR [22,23,24], and loop-mediated isothermal amplification (LAMP) assays [25,26,27]. These methods are rapid, efficient, and accurate in specifically identifying mating types.

Information on the complete life cycle of *B. maydis* populations is beneficial for understanding SCLB epidemics and formulating management strategies because sexual recombination could produce novel genotypes capable of overcoming fungicides or host resistance, thereby generating genetic variation [28,29,30]. However, a crucial issue concerning *B. maydis* is whether it has the potential to undergo sexual genetic recombination as inferred from mating types, pathogenic divergence, and genetic diversity assessed with microsatellite and single-nucleotide polymorphism (SNP) markers [31,32,33,34,35]. In previous studies, researchers revealed the presence of high pathogenicity and abundant genetic diversity in several regional *B. maydis* populations in China [10,16,36]. Because the sexual phase of *B. maydis* has not been discovered under field conditions, investigations of mating type frequencies are conducive to deducing the potential of random mating [37,38]. In comparison, the presence of both MAT1-1 and MAT1-2 isolates is not sufficient to prove the occurrence of sexual recombining [31]; equilibrated mating type distribution frequency suggests the existence of reproducing sexually, with results consistent with random recombination according to equal ratios of mating type in various phytopathogens, such as *Rhynchosporium secalis* [31], *Stagonospora nodorum* [39], *Cercospora sojina* [40], *Diplodia pinea* [41], *Exserohilum turcicum* [42], *Venturia effusa* [43], *Didymella tanaceti* [44], and *Phymatotrichopsis omnivora* [32].

In China, the distribution and fertility of the two mating types of *B. maydis* were determined from various locations in the provinces of Fujian and Sichuan [16,45]; however, little is known about mating types in other regions in China. The main objectives of this investigation were (i) to monitor whether the two mating type populations of *B. maydis* changed under natural conditions during 2015 to 2025 in China and (ii) to determine the distribution and ratio of MAT1-1 and MAT1-2 populations of *B. maydis* across large-scale geographical spatial scales (isolates from provincial regions) and microgeographical spatial scales (within fields and within leaves) to deduce the potential reproductive strategies of *B. maydis* populations in China.

## 2. Materials and Methods

### 2.1. Isolation of B. maydis Isolates

Small leaf pieces (3 mm × 6 mm) from diseased leaves were initially sterilized externally in 75% (*v*/*v*) ethanol for 2 min and in 0.1% (*w*/*v*) HgCl_2_ water solution for 30 s. Thereafter, the pieces were washed three times in sterile ddH_2_O, air-dried on sterilized Miracloth, placed onto potato dextrose agar (PDA; potato 200 g·L^−1^, dextrose 20 g·L^−1^, agar 18 g·L^−1^), and cultured in an incubator in the dark at 28 °C for three days [16]. Pure isolates were acquired via mycelial tip isolation. Briefly, an agar disk (5 mm × 5 mm) with hyphal tips from the margin of a 3-day-old colony was placed onto a fresh PDA plate. The PDA plates were cultured in an incubator in the dark at 28 °C for three to five days. The process was repeated twice per isolate. Finally, pure culture isolates were grown on PDA slants and stored in a freezer at 4 °C for future use.

### 2.2. DNA Extraction

Genomic DNA of each *B. maydis* isolate was extracted from mycelia using a rapid mini-preparation method [46]. Briefly, 5-day-old hyphae of each *B. maydis* isolate were collected using a sterile toothpick and transferred into a 2 mL tube containing 800 μL extraction buffer [2% CTAB (*w*/*v*), 100 mmoL·L^−1^ Tris-HCl (pH 8.0), 20 mmoL·L^−1^ EDTA, 1.4 moL·L^−1^ NaCl, and 10% SDS (*w*/*v*)] and 800 μL chloroform/isoamylol (24:1). The tubes were gently mixed and maintained at 50 °C for 30 min and then centrifuged at 12,000 r·min^−1^ for 20 min. Approximately 600 μL liquid supernatant from each tube was transferred into a fresh 1.5 mL tube containing 600 μL ice-cold isopropanol and deposited at −20 °C for 30 min. DNA precipitates were washed twice with 70% ice-cold ethanol and resuspended with 50 μL cell-free ddH_2_O. The concentration and quality of each DNA sample were assessed using a K5800C spectrophotometer (Kaiao Technology Development Co., Ltd., Beijing, China) and diluted to 50 ng·μL^−1^ using cell-free ddH_2_O. The DNA samples were stored at −20 °C in a freezer for long-term storage.

### 2.3. Mating Type Determination

A multiplex polymerase chain reaction (PCR) with mating type-specific primers (ChMAT01-3 for MAT1-1 and ChMAT02-2 for MAT1-2) was used to identify *B. maydis* mating types [23]. Primer details and PCR amplification procedures are listed in Table 1. Each 25-μL PCR volume contained 12.5 μL 2× Rapid Taq Master Mix (Vazyme, Nanjing, China), 10 pmol of each primer, and 50 ng DNA template. The PCR was carried out in a Thermal Cycler C1000^TM^ System (Bio-Rad Laboratories, Life Science Research, Hercules, CA, USA). PCR assays containing DNA templates from both mating type isolates and reagent only were used as positive and negative controls, respectively. The PCR products were separated via 1.0% agarose gel electrophoresis (Biowest Inc., Loire Valley, France) with ethidium bromide, visualized, and imaged using a Tanon MINI Space 1000 System (Tanon Science & Technology, Shanghai, China).

### 2.4. Temporal Changes in the Two Mating Types in China

Seven fixed sites located in Southeast China were chosen for monitoring the dynamics of the two mating types of *B. maydis* from 2015 to 2025. Six to nine commercial corn fields at each monitoring site were randomly selected for annual sampling. Two to three corn leaves with at least two visible SCLB lesions per leaf were randomly sampled at each field when corn reached the flowering or ripening stage. At least two isolates were isolated from each leaf as described in Section 2.1. A well-growing isolate from each leaf was selected for further use. Finally, 105, 95, 87, 104, 89, 91, 106, 103, 91, 107, and 115 isolates were obtained from 2015 to 2025, respectively. These isolates were grown on PDA slants, stored in a freezer at 4 °C, and used for monitoring the dynamics of the two mating types of *B. maydis* in China.

### 2.5. Large-Scale Geographical Distribution of the Two Mating Types in China

**Figure 1:**
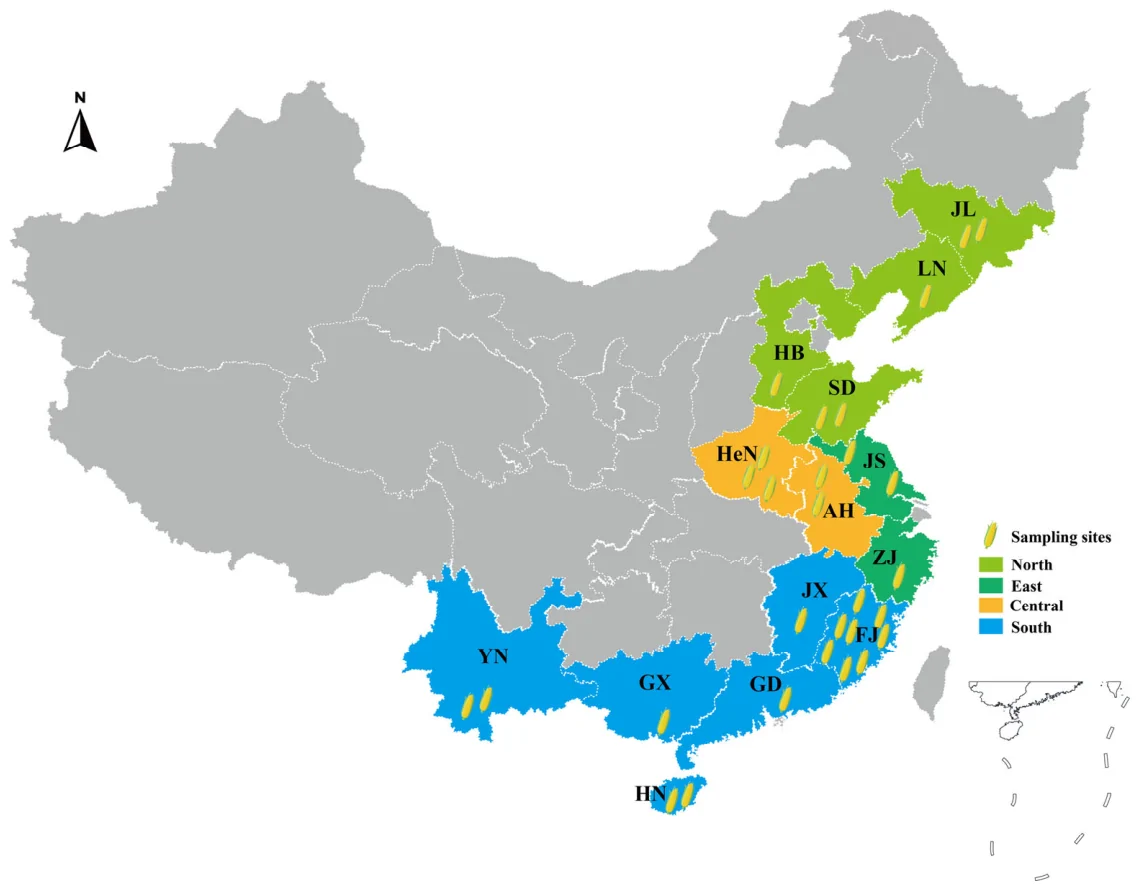
Map of the fourteen provinces in China showing the regions for collection of *Bipolaris maydis* isolates. JL, LN, HB, SD, HeN, AH, JS, ZJ, JX, FJ, GD, GX, YN, and HN indicate that *B. maydis* isolates were collected from the provinces of Jilin, Liaoning, Hebei, Shandong, Henan, Anhui, Jiangsu, Zhejiang, Jiangxi, Fujian, Guangdong, Guangxi, Yunnan, and Hainan in China, respectively.

The mating type distribution and ratio of a large-scale geographical collection of isolates, covering 14 provincial populations in four main corn-growing regions in northern [Jilin (JL), Liaoning (LN), Hebei (HB), and Shandong (SD) Provinces], central [Henan (HeN) and Anhui (AH)], eastern [Jiangsu (JS) and Zhejiang (ZJ)], and southern [Jiangxi (JX), Fujian (FJ), Guangdong (GD), Guangxi (GX), Yunnan (YN), and Hainan (HN)] China (Figure 1), were determined using multiplex PCR as described in Section 2.3. Briefly, one to three corn leaves with at least one typical SCLB lesion per leaf were collected randomly from each field during July to October, and at least 10 commercial corn fields were sampled in each province. A well-growing isolate was taken from each leaf as described in Section 2.1. These isolates were used to determine the distribution of mating types of *B. maydis* in China.

### 2.6. Microgeographical Distribution of the Two Mating Types Within Fields

The microgeographical distribution and ratio of mating types within fields representing 14 provincial populations (JL, LN, HB, SD, HeN, AH, JS, ZJ, JX, FJ, GD, GX, YN, and HN Provinces) in China were identified using multiplex PCR as described in Section 2.3. Briefly, a heavily infected field in each province was selected for sampling, and 24 or 36 corn leaves with at least one typical SCLB lesion per leaf were randomly collected from each field during July to October. A well-growing isolate was taken from each leaf as described in Section 2.1. These isolates were used to determine the distribution of mating types of *B. maydis* within fields.

### 2.7. Microregional Distribution of the Two Mating Types Within Leaves

The microregional distributions and ratios of mating types within leaves across 14 major corn-growing provinces (JL, LN, HB, SD, HeN, AH, JS, ZJ, JX, FJ, GD, GX, YN, and HN) in China were determined using multiplex PCR as mentioned in Section 2.3. Briefly, a heavily infected leaf (>15 lesions) was randomly collected from each province during July to October. All visible SCLB lesions were excised from each leaf and used for isolation of *B. maydis* isolates as described in Section 2.1. These isolates were used for determination of the distribution of mating types of *B. maydis* within leaves.

### 2.8. Data Analysis

Pearson Chi-square tests for goodness of fit at *p* < 0.05 were performed in DPS software v7.05 (RuiFeng Information Technology Co. Ltd., Hangzhou, China) to determine whether the observed proportions of the two mating type isolates within individual subpopulations (different regions, fields, and leaves) significantly deviate from a null hypothesis ratio of 1:1 [47].

## 3. Results

### 3.1. Temporal Changes in the Two Mating Types in China

**Figure 2:**
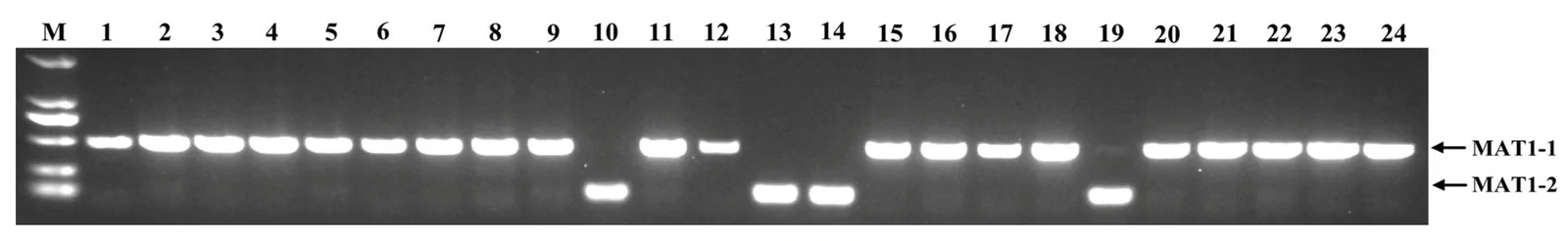
Multiplex PCR amplification of the two mating types of twenty-four representative *Bipolaris maydis* isolates with specific primers ChMAT01-3 and ChMAT02-2. M: 2 kb DNA ladder. Lanes 1–9, 11–12, 15–18, and 20–24 show MAT1-1 isolates of *B. maydis*, and lanes 10, 13–14, and 19 represent MAT1-2 isolates, respectively. A specific amplicon of either 490 bp or 136 bp was amplified for an MAT1-1 or MAT1-2 isolate.

**Figure 3:**
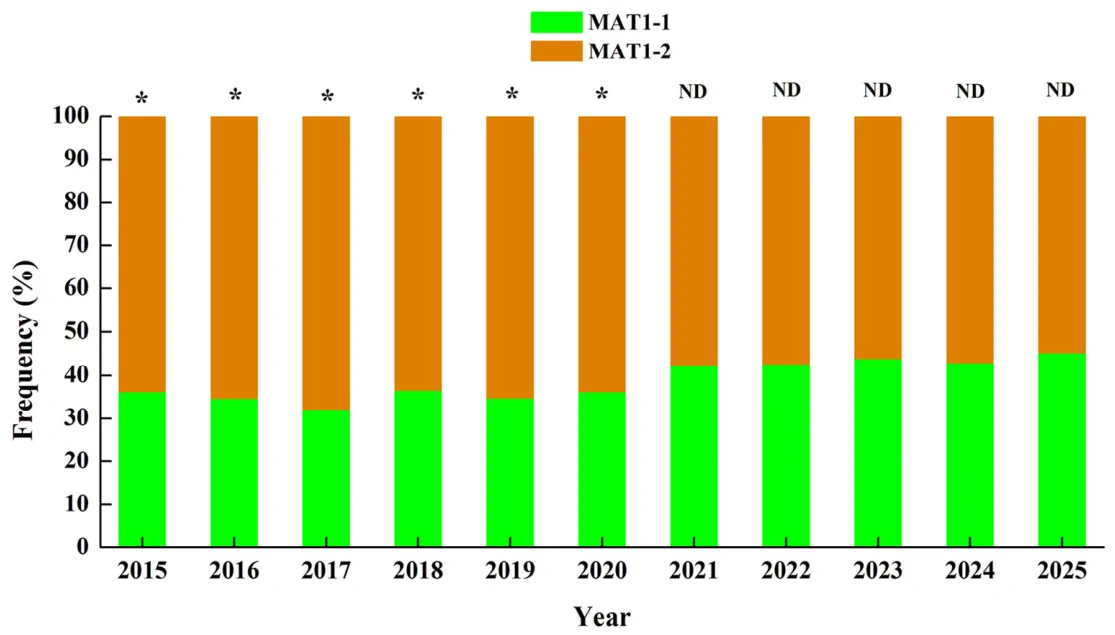
Temporal changes in the two mating types of *Bipolaris maydis* from 2015 to 2025 in China. Pearson Chi-square tests were conducted using DPS software v7.05 (Hangzhou Reifeng Information Technology Ltd., Hangzhou, China). *: *p* < 0.05; ND: *p* > 0.05 denotes an equal 1:1 mating type ratio.

A total of 1093 isolates of *B. maydis* were recovered from seven fixed monitoring sites in Southeast China from 2015 to 2025. The mating type of each isolate was successfully determined using multiplex PCR with mating type-specific primers (Figure 2). Generally, the MAT1-1 and MAT1-2 isolates of *B. maydis* co-existed throughout the seven monitoring sites from 2015 to 2025, whereby the temporal dynamics of the two mating types were skewed toward MAT1-2, with frequencies of more than 54% (Figure 3). The frequencies of the two mating types deviated significantly (*p* < 0.05) from the null hypothesis of a 1:1 ratio from 2015 to 2020, while the proportions of the two mating types tended toward a 1:1 ratio (*p* > 0.05) from 2021 to 2025 (Figure 3), implying that the two populations gradually approached equilibrium and that random mating may have occurred under natural conditions.

### 3.2. Large-Scale Geographical Distribution of the Two Mating Types in China

**Figure 4:**
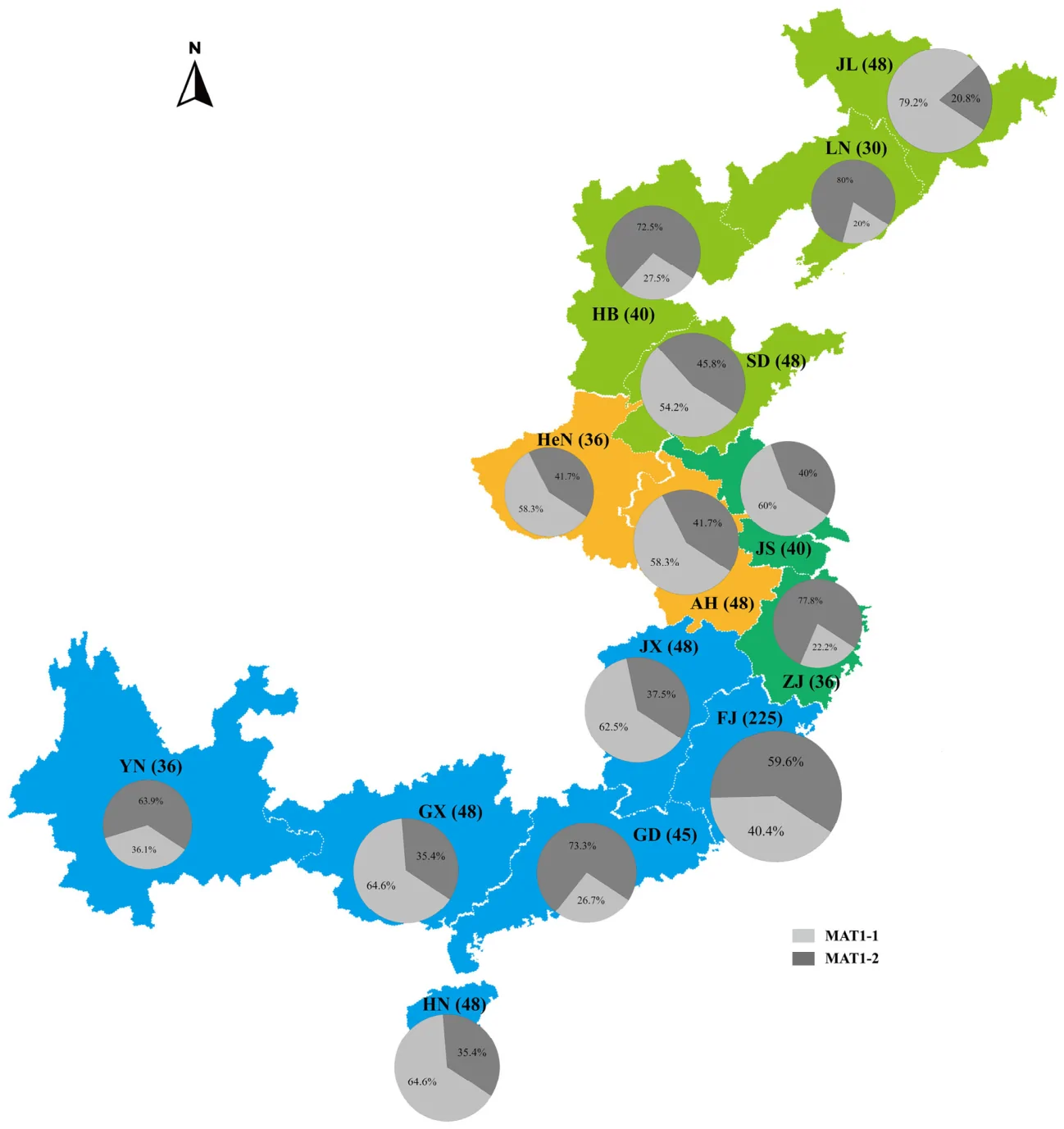
Large-scale geographical distribution of the two mating types of *Bipolaris maydis* in China. JL, LN, HB, SD, HeN, AH, JS, ZJ, JX, FJ, GD, GX, YN, and HN indicate that *B. maydis* isolates were collected from the provinces of Jilin, Liaoning, Hebei, Shandong, Henan, Anhui, Jiangsu, Zhejiang, Jiangxi, Fujian, Guangdong, Guangxi, Yunnan, and Hainan in China, respectively. The numbers in parentheses represent the number of *B. maydis* isolates.

Overall, both MAT1-1 and MAT1-2 isolates were detected in each province of China, with 47.7% (370 isolates) of isolates being identified as MAT1-1 and 52.3% (406) identified as MAT1-2 (Figure 4). Mating type frequencies in six provincial populations (JL, LN, HB, FJ, GD, and ZJ) skewed significantly (*p* < 0.05) from the 1:1 ratio according to Pearson Chi-square tests, and the other eight provincial populations (SD, HeN, AH, JS, JX, GX, YN, and HN Provinces) showed no significant differences (*p* > 0.05) in mating type ratios (Figure 4).

### 3.3. Microgeographical Distribution of the Two Mating Types Within Fields

In total, the two opposite mating type isolates were identified from each field, with 49.7% (197 isolates) of isolates being identified as MAT1-1 and 50.3% (199) identified as MAT1-2 (Table 2). A significant unsymmetrical (*p* < 0.05) mating type distribution was observed in four field populations (JL, LN, ZJ, and GD), with a significant skew toward MAT1-2 being observed in LN, ZJ, and GD populations and a skew toward MAT1-1 observed in the JL population (Table 2). The mating type frequencies in the other ten field populations (HB, SD, HeN, AH, JS, JX, FJ, GX, YN, and HN) were consistent with a 1:1 ratio based on Pearson Chi-square tests (Table 2).

### 3.4. Microregional Distribution of the Two Mating Types Within Leaves

In all of the cases examined, both MAT1-1 and MAT1-2 isolates were recovered from each single leaf (Table 3). In total, 168 isolates were verified as MAT1-1 and 149 isolates as MAT1-2 (Table 3). A significant unequal (*p* < 0.05) mating type distribution was observed in four leaf populations (JL, ZJ, FJ, and GD), with a significant skew toward MAT1-1 being observed in JL and GD populations, as well as a skew toward MAT1-2 being observed in ZJ and FJ (Table 3). The mating type frequencies in the other ten leaf populations (LN, HB, SD, HeN, AH, JS, JX, GX, YN, and HN) were consistent with a 1:1 ratio (Table 3).

## 4. Discussion

To date, the sexual cycle in natural *B. maydis* populations has yet to be discovered in various environments (e.g., different regions, within fields and leaves) [16,45], whereas sexual reproduction is universally believed to offer a potential avenue for generating genetic diversity in fungal pathogens [2,35,48]. In the present study, we comprehensively investigated the temporal dynamics and distribution of mating types in *B. maydis* populations using multiplex PCR with mating type-specific primers. The results showed that both MAT1-1 and MAT1-2 isolates were detected throughout the monitoring period at large-scale geographical monitoring locations, despite *B. maydis* populations among year groups being mostly dominated by the MAT1-2 population. The results of Pearson Chi-square tests showed that mating type frequencies were significantly skewed (*p* < 0.05) toward the MAT1-2 population from 2015 to 2020 and practically symmetric after 2020 (Figure 2). Our findings suggest that Chinese natural *B. maydis* populations may meet the spatiotemporal conditions required for sexual reproduction [31].

The co-existence of both MAT1-1 and MAT1-2 isolates does not provide sufficient evidence to prove the occurrence of sexual reproduction [31]. An approximately 1:1 ratio of the opposite mating types throughout large spatial scales frequently suggests the presence of sexual reproduction according to the hypothesis of frequency-dependent selection [38]. The observed equal distribution could also be explained by gene drift, which disperses MAT1-1 and MAT1-2 isolates across the regional populations. The proportion of the two mating type idiomorphs was established at various spatial scales in major corn-growing provinces in China (1489 isolates). The results indicated that mating type ratios were balanced in most provincial populations. Consequently, the results provide convincing evidence supporting acceptance of the null hypothesis of equalization in general, which is in accordance with frequency-dependent selection [38]. The asymmetric mating type distributions were also detected occasionally in populations, which have been reported previously in other fungal plant pathogens [42,49,50,51,52,53]. Therefore, the expected 1:1 frequency between the two mating type populations may facilitate opportunities for mating in *B. maydis*. The findings of the present study are consistent with those reported for numerous heterothallic plant pathogens, such as *R. secalis* on barley [31], *S. nodorum* on wheat [39], *C. sojina* on soybean [40], *V. effusa* on pecan [43], *D. tanaceti* on pyrethrum [44], *Phyllosticta citricarpa* on lemon [54], *V. carpophila* on peach [55], and *P. omnivora* on cotton and alfalfa [32], where mating type distributions and frequencies have been surveyed, and cross-interactions have been confirmed to occur. In addition, several *B. maydis* populations in China are known to be characterized by a high level of genetic diversity despite occasional presence of linkage disequilibrium [2,10,36].

In this study, six provincial populations of *B. maydis* (JL, LN, HB, FJ, GD, and ZJ) exhibited mating type proportions that significantly deviated from a 1:1 ratio. A remarkable deviation from a 1:1 ratio was recorded between MAT1-1 and MAT1-2 populations. Two primary factors may explain this phenomenon. Firstly, extrinsic selection, such as host resistance or fungicide spraying, may favor one idiomorph over the other, thereby impacting the proportion of mating types [39]. Secondly, mating type loci may play other important functions in asexual processes [56,57,58], pathogenicity [57,58,59], and secondary metabolism [60,61,62].

Concerning the spatiotemporal distribution of mating types in heterothallic fungal plant pathogens, it is frequently dominated by one mating type in natural populations. Li demonstrated that the mating type distribution of the broad-host-range causal agent, *Calonectria pauciramosa*, in nine countries was predominantly MAT1-2 [63], and Yan demonstrated that the mating type of the rice blast pathogen *Magnaporthe oryzae* was significantly skewed toward MAT1-2 in China [53]. During the 2017 to 2020 cropping seasons, Le and Nguyen reported an asymmetric temporal distribution of the two mating types of *Berkeleyomyces rouxiae*, resulting in black root rot on cotton in New South Wales, Australia [50]. This unequal mating type distribution has also been observed in *B. maydis* populations from Fujian Province, China, and Australia [16,64]. In this study, following several isolate collections, both MAT1-1 and MAT1-2 isolates were frequently detected in each field or even in a single leaf from all sampled provinces across a large-scale geographical distribution, which signifies that the physical presence of opposite mating types in natural conditions is widespread. In addition, the presence of the two mating types in an equal distribution was also observed in these fields or even in a single leaf. This close proximity and observed uniform distribution of the two mating types is consistent with the possibility of sexual recombination [65].

In the present study, adequately large isolate numbers (2582 isolates) and multi-dimensional sample collections throughout diverse environments and regions in China were employed to strengthen the reliability of the data. The results showed that both MAT1-1 and MAT1-2 isolates were detected at all spatiotemporal scales tested. Nonetheless, no sexual isolates of *B. maydis* were identified in this study. Several factors may explain why no sexually reproducing isolates were detected among the *B. maydis* isolates collected from corn. One possible explanation as to why sexual isolates have yet to be detected for *B. maydis* could be attributed to sexual reproduction occurring on alternate hosts. Many previous reports have verified that numerous heterothallic fungi can complete their sexual cycle on alternate hosts [66,67,68,69]. It is likely that *B. maydis* may complete sexual reproduction on extensively distributed gramineous weeds. Secondly, another explanation for the failure to discover the sexual morph in the present study is the fact that the sampling period may not have coincided with the appropriate stage of the disease cycle, as sexual reproduction typically occurs on senescent leaves at the later stage of hosts [39]. Lastly, it has been suggested that sexual reproduction could be interfered with by inadaptable environmental conditions or mutations in other genes involved in the regulation of sexual processes [39]. Further studies are necessary to focus on surveying alternate hosts in nature, given the crucial role played by alternate hosts in the pathogen’s sexual cycle. Furthermore, sexual morphs of *B. maydis* should also be surveyed in the field, as, at present, little is known regarding sexual reproduction in *B. maydis*.

## 5. Conclusions

In conclusion, both mating types were found to co-occur throughout the entire monitoring period and across all locations, with mating types being predominantly characterized by MAT1-2 from 2015 to 2020 and approximately equal after 2020, implying that the two populations exhibited a tendency toward equilibrium. Our results demonstrate the detection of both mating types in equal ratios and occupying the same leaf or field, suggesting frequency-dependent selection consistent with sexual reproduction. Overall, our results, combined with those from previous reports involving high genetic diversity, provide indirect evidence supporting the potential for sexual reproduction in *B. maydis* in nature.

## Figures and Tables

**Table 1:** Primers used in this study, including primer code, sequence, product size, target region, and PCR amplification procedure.

Primer Code	Primer Sequence (5′-3′)	Product Size (bp)	Target Region	PCR Amplification Procedure
ChMAT01-3	F: ACCACGAGACAACATACGC	490	MAT1-1	95 °C 3 min; 35 cycles of 95 °C 15 s, 55 °C 15 s, 72 °C 30 s; 72 °C 5 min; 16 °C for temporary preservation
	R: GTTTGAGGTGAGGGCAGA			
ChMAT02-2	F: GGCGAAGTTTGTGGGTTT	136	MAT1-2	
	R: GCGATGTCTGGGCTGATT			

**Table 2:** Distributions and ratios of the two mating types of *Bipolaris maydis* on a small provincial scale within individual fields.

Locations	Populations	Number of Isolates	Year Collected	MAT1-1:MAT1-2	Pearson Chi-Square Tests ^1^	
					*χ*^2^ Value	*p* Value
North	Jilin	24	2024	21:3	12.04	<0.001
	Liaoning	24	2023	5:19	7.04	0.008
	Hebei	36	2022	16:20	0.25	0.617
	Shandong	36	2020	19:17	0.03	0.868
Central	Henan	36	2020	20:16	0.25	0.617
	Anhui	36	2018	21:15	0.69	0.405
East	Jiangsu	24	2019	12:12	0.04	0.838
	Zhejiang	24	2018	4:20	9.36	0.002
South	Jiangxi	24	2019	14:10	0.38	0.540
	Fujian	36	2025	21:15	0.69	0.405
	Yunnan	24	2022	11:13	0.04	0.838
	Guangxi	24	2019	17:7	3.38	0.066
	Guangdong	24	2018	3:21	12.04	<0.001
	Hainan	24	2019	13:11	0.04	0.838
Total		396	2018–2025	197:199	<0.01	0.960

^1^ Pearson Chi-square tests were conducted using DPS software (v7.05, Hangzhou Reifeng Information Technology Ltd., Hangzhou, China). *p* > 0.05 denotes an equal 1:1 mating type ratio.

**Table 3:** Distributions and ratios of the two mating types of *Bipolaris maydis* on a small provincial scale within individual leaves.

Locations	Populations	Number of Isolates	Year Collected	MAT1-1:MAT1-2	Pearson Chi-Square Tests ^1^	
					*χ*^2^ Value	*p* Value
North	Jilin	26	2024	24:2	16.96	<0.001
	Liaoning	17	2023	4:13	3.77	0.052
	Hebei	25	2022	9:16	1.44	0.230
	Shandong	24	2020	12:12	0.04	0.838
Central	Henan	24	2020	14:10	0.38	0.540
	Anhui	24	2018	12:12	0.04	0.838
East	Jiangsu	24	2019	13:11	0.04	0.838
	Zhejiang	19	2018	2:17	10.32	0.001
South	Jiangxi	23	2019	15:8	1.57	0.211
	Fujian	24	2017	5:19	7.04	0.008
	Yunnan	20	2022	8:12	0.45	0.502
	Guangxi	21	2019	15:6	3.05	0.081
	Guangdong	25	2018	21:4	10.24	0.001
	Hainan	21	2019	14:7	1.71	0.190
Total		317	2017–2024	168:149	1.02	0.312

^1^ Pearson Chi-square tests were conducted using DPS software (v7.05, Hangzhou Reifeng Information Technology Ltd., Hangzhou, China). *p* > 0.05 denotes an equal 1:1 mating type ratio.

## Data Availability

The original data in the study are included in the article. Further inquiries can be directed to the corresponding authors.
